# Effectiveness of an educational intervention about inhalation technique in healthcare professionals in primary care: a cluster randomized trial

**DOI:** 10.3389/fphar.2023.1266095

**Published:** 2023-10-17

**Authors:** Noemí Vázquez-González, José Leiva-Fernández, Víctor M. Cotta-Luque, Francisca Leiva-Fernández, Francisca Rius-Díaz, Francisco Martos-Crespo, Elisa Martín-Montañez, Pilar Barnestein-Fonseca

**Affiliations:** ^1^ Department of Pharmacology and Pediatrics, Faculty of Medicine, University of Malaga. IBIMA Plataforma BIONAND, Malaga, Spain; ^2^ Transfusion, Tissues and Cells Centre of Malaga, Andalusian Health Services, Malaga, Spain; ^3^ UGC Velez Sur Area Sanitaria Malaga Este-Axarquía, Malaga, Spain; ^4^ Multiprofessional Teaching Unit of Community and Family Care Primary Care District Malaga-Guadalhorce Knowledge Management Unit Malaga-Guadalhorce Health District, Andalusian Health Services, IBIMA Plataforma BIONAND, Malaga, Spain; ^5^ Department of Epidemiology and Public Health, Faculty of Medicine, University of Malaga, Malaga, Spain; ^6^ Research Unit, Instituto CUDECA de Estudios e Investigación en Cuidados Paliativos, IBIMA Plataforma BIONAND, Málaga, Spain

**Keywords:** chronic obstructive pulmonary disease, inhalation technique, educational intervention, primary care, healthcare professionals, cluster randomized controlled trial

## Abstract

**Background:** Incorrect inhalation technique (IT) is an important issue for chronic obstructive pulmonary disease (COPD) patients and healthcare professionals. Studies in which counseling is carried out with healthcare professionals beforehand so that they can properly educate their patients are required. The objective of the present trial is to assess the improvement in the performance of the IT in subjects with COPD and prescribed inhaled therapy after the implementation of an educational intervention conducted by their general practitioners.

**Methods:** A cluster randomized clinical trial was conducted. A total of 286 COPD patients received scheduled inhalation therapy from 27 general practices in seven primary care centers. A teach-back educational intervention was implemented for both healthcare professionals and patients. The primary outcome of this study was the performance of the correct inhalation technique. It is considered a good technique if all steps in the inhalation data sheet are correctly performed. The secondary outcomes were assessed using forced spirometry, the basal dyspnea index, the Medical Research Council dyspnea scale, St George’s Respiratory Questionnaire (SGRQ), and EuroQoL5D-5L for health-related quality of life. A one-year follow-up was conducted using an intention-to-treat analysis.

**Results:** After the intervention, incorrect IT was observed in 92% of professionals and patients, with rates reaching 50% and 69.2%, respectively. The effectiveness in patients was significant, with a number needed to treat of 2.14 (95% CI 1.79–2.66). Factors related to correct IT in patients included the type of intervention, length of intervention (>25 min), good pulmonary function, age (youngest <=65, oldest >83), and less limitation of activity due to dyspnea. There was no relation with the cluster.

**Conclusion:** This study shows the effectiveness of direct inhaler technique training provided by a trained professional on an appropriate timescale (for example, a specific consultation for medication reviews), aiming to help subjects improve their performance using the teach-back method. This could be an encouraging intervention to improve medication adherence and health promotion in people with COPD.

**Clinical Trial Registration:** clinicaltrials.gov, identifier ISRCTN93725230.

## 1 Introduction

Medication adherence, referring to the level of participation in terms of individuals taking medications as prescribed, is known to be a central health problem, especially important in the treatment of chronic diseases. After half a century of adherence-related research and increased knowledge of the factors involved in its implementation (>200), adherence rates remain fairly stable (Vrijens et al., 2012; Conn and Ruppar, 2017; Ellis et al., 2023). Thus, although the rates observed in clinical trials are considered to be very high (70%–90%), they vary between 10% and 40% in clinical practice (WHO, 2015; Bhattarai et al., 2020). Adherence to medication is essential for individuals to receive the potential therapeutic benefits of the prescription, especially in those conditions where the application of the treatment is much more complex than taking a pill. Lack of adherence is associated with considerable morbidity/mortality (Ellis et al., 2023).

Chronic obstructive pulmonary disease (COPD) is among the significant health challenges worldwide (Duarte-de-Araújo et al., 2019; Lindh et al., 2019) due to its high prevalence (11.7%) (Padmanabhan et al., 2019), the high healthcare costs it leads to (Barja-Martínez et al., 2019; Padmanabhan et al., 2019; Rincon-Montaña and Rosselli, 2019), and the negative effects it has on the quality of life (Rincon-Montaña and Rosselli, 2019). The treatment relies primarily on inhaled medication (Barja-Martínez et al., 2019; Duarte-de-Araújo et al., 2019; Padmanabhan et al., 2019) through currently available devices, which include dry powder inhalers (DPIs), metered-dose inhalers (MDIs), pressurized metered-dose inhalers (pMDIs), and soft mist inhalers (SMIs) (Rincon-Montaña and Rosselli, 2019; Dekhuijzen et al., 2022; Lindh et al., 2022). These devices require many steps, which may be complex, making them difficult to use (Melzer et al., 2017; Takaku et al., 2017; Dekhuijzen et al., 2022). Therefore, the incorrect use of these devices becomes a major problem, leading to reduced therapeutic effects, increased symptoms, and ineffective disease control (Vila Jato, 2020; Dekhuijzen et al., 2022). Increased hospitalization, emergency room visits, and the need for antibiotics and corticosteroids have also been reported, increasing the cost of the disease and leading to adverse effects and a reduction in therapeutic alternatives (Vila Jato, 2020; Dekhuijzen et al., 2022).

To acquire proficiency in handling inhalers, it is essential to provide proper training to patients (Poureslami et al., 2016; Dhadge et al., 2020; Luley et al., 2020; Rodrigues et al., 2021; Lindh et al., 2022; Sulku et al., 2022), since education on the proper use of inhalers is received by only a small number of them (Poureslami et al., 2016; Dekhuijzen et al., 2022).

Furthermore, COPD clinical practice guidelines pay particular attention to proper advice and instruction on inhaler management as a vital part of treatment. They state that the first time a device is prescribed, the IT should be explained and a demonstration should be performed for the patient, training the patient as many times as necessary to achieve a proper IT and confirming that the patient can use it properly. Subsequently, the patient should demonstrate, with their own device, how they perform the IT at each visit to ensure successful execution (Andaluz de Salud, 2019; Global Iniciative for COPD, 2022; Miravitlles et al., 2022). The accessibility of patients to healthcare providers is important for a correct IT because professionals will have more opportunities to assess the patient periodically and train them appropriately (Yawn et al., 2012).

Healthcare professionals (e.g., general practitioners, respiratory physiotherapists, community pharmacists, or health educators) also need to be properly trained, as evidence suggests that their knowledge of the use of inhaled medicines can be improved (Al-Otaibi, 2020; Cvetkovski et al., 2020). IT also improves for them after training, either by attending educational workshops (Al-Otaibi, 2020) or conferences (Takemura et al., 2011), providing explanatory leaflets (Cvetkovski et al., 2020; Matsuyama et al., 2022), or demonstrations with placebo devices (Basheti et al., 2008) or through videos (Cvetkovski et al., 2020; Matsuyama et al., 2022).

This highlights the importance of conducting studies in which the educational intervention is carried out beforehand with public and community health personnel so that they can properly counsel their patients. To the best of our knowledge, no trial of educational intervention concerning the IT has been carried out among general practitioners (GPs) to assess its effectiveness on COPD patient IT performance. Therefore, this study aimed to implement a cluster randomized trial among healthcare professionals at primary care centers (PCCs) to evaluate the effectiveness of an educational intervention on the improvement in the performance of the IT in patients with COPD and prescribed inhaled therapy after the implementation of this educational intervention with their GPs.

## 2 Materials and methods

### 2.1 Study design

A pragmatic cluster randomized controlled trial (ISRCTN93725230) was conducted. The cluster has a two-level design: at the higher or second level is the GP (the recipient of the educational intervention), and at the lower or first level are the patients (who provided consent for their participation and received the educational intervention from their GP). The PROF-EPOC trial gained approval from the Malaga Provincial Ethical Committee (12/12/13). The protocol of the study was broadly described by Leiva-Fernández et al. (2016). We adhered to the CONSORT reporting guidelines (Schulz et al., 2010).

### 2.2 Setting, participants, recruitment, and follow-up

A total of 286 patients with a diagnosis of COPD who were receiving scheduled inhalation therapy were chosen by a non-random consecutive sampling method from 27 general practices in seven PCCs in Málaga and Almería, Spain.

The sample size was determined to detect a 25% difference in the percentage of the correct IT between the groups, aiming for a statistical power of 80%, confidence level of 95%, and design effect (DE) of 2.3 (Christie et al., 2009; Bunker et al., 2012; Thompson et al., 2012). A potential loss of 40% was estimated.

The inclusion criteria were as follows: patients with a COPD diagnosis receiving clinical attention at the PCC included in the trial, those who had been prescribed scheduled inhalation treatment, and those who had given their consent to participate in the trial by signing an informed consent form. The exclusion criteria were the presence of another respiratory illness not included in the definition of COPD and cognitive impairments that make it impossible for the individual to complete the outcome questionnaires or fully engage with the educational intervention. These criteria were all ascertained from the patient’s clinical record.

The GPs included were required to be physicians caring for patients included in the COPD Process of the Andalusian Health Service Guidelines (COPD PAI) (Andaluz de Salud, 2019) and who had signed the informed consent form. The exclusion criteria were reluctance to participate or leaving the job during the trial.

Twenty-seven GPs were chosen using a non-probabilistic consecutive sampling method: 14 GPs were used as the control group (CG) and 13 as the intervention group (IG). GPs were invited to participate and randomized to one of the two groups using a block randomization technique. The GP’s baseline visit was undertaken once the randomization had been completed. The study variables were collected during this time, and the IT of the various inhalers (Handihaler^®^, Accuhaler^®^, Turbuhaler^®^, Breezhaler^®^, and pressurized metered-dose inhalers) was assessed through a step-by-step test that was specifically designed for the study, following guidelines of the Spanish Society of Pneumology and Thoracic Surgery (SEPAR, its acronym in Spanish) (Vila Jato, 2020).

Patients were contacted by telephone and asked to participate. An appointment was then arranged at their healthcare center. This first appointment (an inclusion visit) involved patients being given more detailed information about the study. If they then agreed to participate, the written consent form would be signed and a baseline visit carried out, where all the variables were measured (this included the assessment of the IT of the various inhalers they used). As for GPs, two groups of patients were established, depending on the group their GP was assigned to, the IG or CG.

A one-year follow-up was conducted after the initial visit. At the final visit (12 months), all variables were collected again, including the IT with all devices in patients and GPs. All measurements were performed by a research assistant who was unaware of the group to which the study subjects were assigned. For the IG subjects (patients), their GPs visited to reinforce IT at 3 and 6 months.

### 2.3 Interventions

#### 2.3.1 GPs

GPs in the IG received group training (2–4 GPs) from the research team with a demonstration of the correct IT per device and the rationale for it. Participants were asked to show their technique with placebo inhalers. Then, using the teach-back method, they were asked to talk about the problems and errors they might have perceived with the technique, and they were then shown the proper technique with each device, in stages, with an explanation that included the importance of each one. Finally, GPs asked questions and practiced the techniques until they achieved good performance.

GPs in the CG were asked to show their technique, but there was no further intervention from the researcher other than correcting critical errors (this is known as a rescue mechanism). A critical error was defined as one that would considerably reduce the deposition of the drug in the lungs (Melani et al., 2017). No other educational intervention was carried out.

#### 2.3.2 Patients

Subjects included in the IG were asked to show their technique with placebo inhalers by their GP. The GP, via the teach-back method, would then ask about the problems and apparent errors with the technique before demonstrating the proper technique with the various devices, step by step, explaining the importance of each. Finally, patients were encouraged to ask questions and practice the techniques until they were performed correctly or until they became tired. Follow-up visits reviewed the IT, corrected errors, and cleared up any doubts. The main purpose at this stage was for patients to identify errors and to give as many demonstrations as necessary to remind them of the proper technique. GPs scheduled patients for follow-up IT visits at 3 and 6 months after the initial visit.

The CG patients had their usual care without any reinforcement interventions.

### 2.4 Outcomes

#### 2.4.1 Individual variables/first-level variables (patients)

The primary outcome was the performance of the correct IT by patients (this was assessed via a step-by-step test designed specifically for each inhaler). This test was designed especially for this study based on SEPAR recommendations (Supplementary Material S1) (SEPAR, 2017; Vila Jato, 2020). It is considered that the IT was appropriate if all the steps for the device were performed correctly.

The step template designed consists of two parts:

• First part: Steps necessary for a correct inhalation technique for each of the devices studied (Handihaler^®^, Accuhaler^®^, Turbuhaler^®^, Breezhaler^®^, and Metered Dose Inhalers), considering several attempts for the patient to perform the inhalation technique. The steps that the patient does not perform correctly in each attempt are marked with a cross.
• Second part: The so-called critical errors have been marked with an asterisk (*). These errors are corrected and considered a rescue mechanism. They are noted as an incidence to be taken into account in the data analysis.

It is considered a good technique if all steps in the inhalation data sheet are correctly performed.

Secondary outcomes included functional status, which was measured by forced spirometry (Garcia-Rio et al., 2013), the dyspnea index, measured using the basal dyspnea index (BDI) (Mahler et al., 1984), and the modified Medical Research Council dyspnea scale (mMRC) (Devon and Holman, 1966). The St George’s Respiratory Questionnaire (SGRQ) (Ferrer et al., 1996) and EuroQoL5D-5L (Herdman et al., 2001) were used to measure health-related quality of life.

The SGRQ is a disease-specific instrument designed to measure the impact on overall health, daily life, and perceived wellbeing in patients with obstructive airway disease. It is sufficiently sensitive to reflect changes in disease activity (Ferrer et al., 1996) (Supplementary Material S2). It should preferably be self-administered, but administration by personal interview is also acceptable. Scores range from 0 to 100, with higher scores indicating more limitations.

The independent variables are age, sex, level of education, inspiratory peak flow, smoking history (patient-reported smoking habit and the number of packs per year), comorbidities, time since the diagnosis of COPD, number of exacerbations/year, total visits to health centers and visits because of COPD, mental and/or cognitive status (Mini-Mental State Examination (MMSE) (Folstein et al., 2000; Lobo et al., 2002)), types of inhalers, previous training in the use of the technique, types of errors in the technique, clinical significance of failure (CSF) (Melani, 2021), time for inhaler training (including a test of how the IT is performed on all devices used by the patient), and prescribed treatment for COPD.

#### 2.4.2 Group variables/second-level variables (GPs)

Group and second-level variables were the correct performance of the ITs by GPs (measured using a step-by-step test specific to each inhaler, the same as that used for patients) and knowledge about COPD and its treatment (assessed with a questionnaire designed especially for this study, based on COPD PAI (Andaluz de Salud, 2019), the Spanish COPD Guidelines (Miravitlles et al., 2022), and the GOLD guidelines (GOLD Report, 2022)).

The independent variables were age, sex, level of education, and access to the COPD clinical practice guidelines.

### 2.5 Statistical analysis

The analysis used an intention-to-treat procedure, considering all patients who were randomized, regardless of what happened during follow-up. For the primary outcome variable, lost data were handled using the worst-case scenario, assuming that the control group losses performed the IT correctly, while the intervention group losses performed the IT incorrectly. For the other variables, a multivariate imputation was performed.

A descriptive statistical analysis was carried out for each of the study variables. The mean and standard deviations were calculated for the quantitative variables, while the absolute and relative frequencies were evaluated for the qualitative variables. The univariate analyses included the following comparisons: an inter-group comparison at baseline, a comparison between the initial and final samples (aimed at assessing the impact of losses on sample structure), and a comparison between the intervention and control branches at the 12-month follow-up. This was conducted with an analysis of variance (ANOVA) or chi-square test, as applicable. The relative risk reduction (RRR), absolute risk reduction (ARR), and number needed to treat (NNT) were calculated with a confidence interval (CI) of 95%.

Multivariate analyses: A logistic regression model was used to analyze the primary outcome (proper IT at 12 months), with the intervention held to be the predictive variable and adjusting for independent variables as modifying factors of the effect of the intervention. A classification tree based on the Chi-square Automatic Interaction Detector (CHAID) technique (Kass, 1980) was made for the main outcome—correctly performing the IT—with all independent variables showing a statistically significant relationship with the dependent variable in the bivariate analysis and/or those included in the study hypothesis or those the literature deems to be clinically relevant. Blocks of variables were established by fields of study (GP and cohort) and sociodemographic variables: age, sex, educational level, and MMSE. There are three blocks with variables related to IT: performance of IT with each device, previous instruction for IT, time since receiving it, who gave the instruction (primary care physician, pulmonologist, community nurse, and community pharmacist), how the instruction was given (demonstration with or without a device and explanation with or without a device or by handout), and quality of life (EuroQol-5D-5L and SGRQ); variables related to functional status: spirometry pattern, severity, % FEV1, and dyspnea (BDI and mMRC) including the time of intervention.

A 5% significance level (*α* = 0.05) and the SPSS statistical package, version 25.0, were used to run the aforementioned analysis.

## 3 Results

### 3.1 Participant recruitment and follow-up

Various health areas of Andalusian Health Services were contacted to recruit GPs from different healthcare centers. Ultimately, 27 GPs were recruited. In total, 1,958 possible participants, identified through health records of the participating GPs in the study, were approached. Finally, 286 patients participated.

**FIGURE 1:**
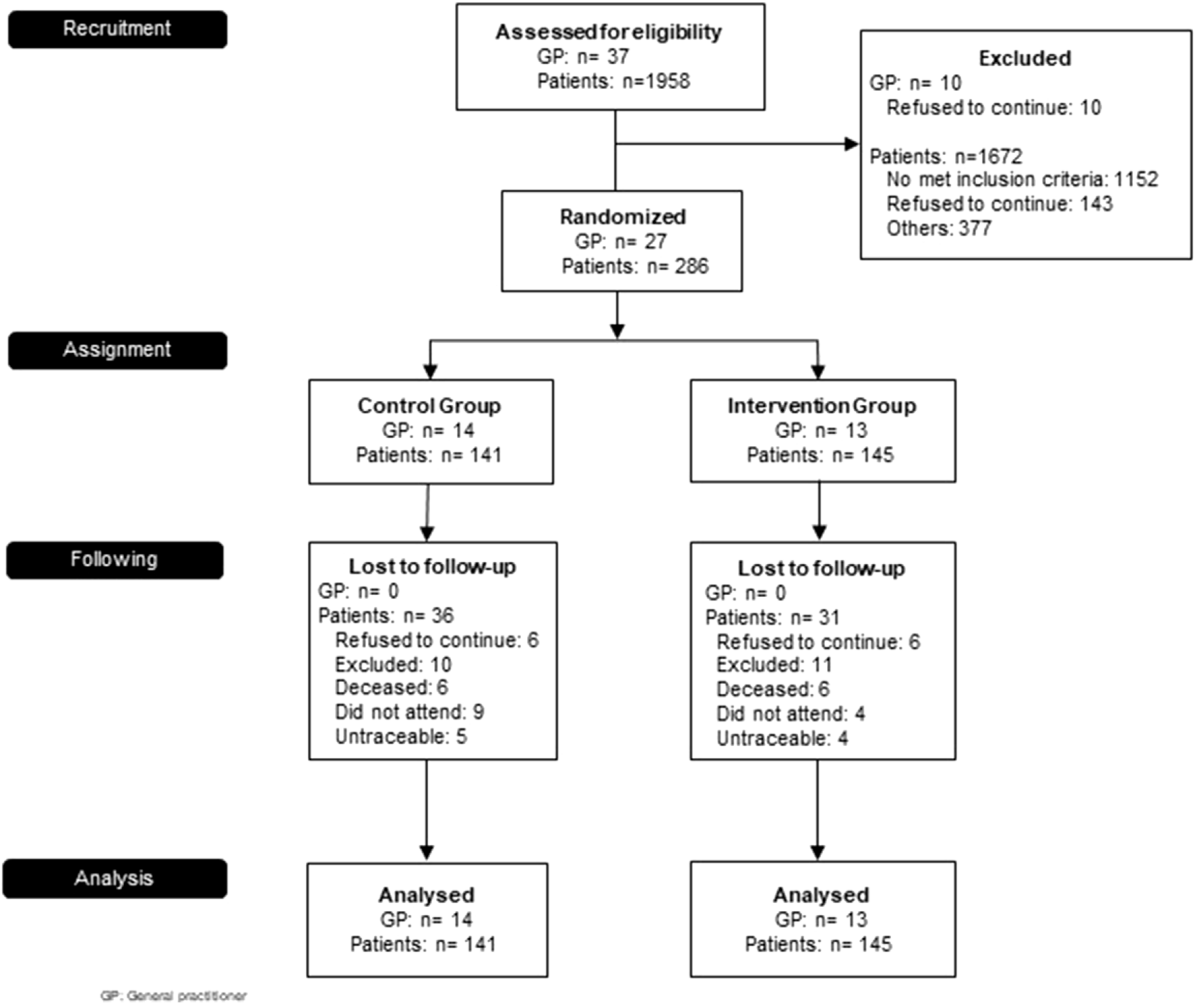
PROF-EPOC CONSORT Flow Diagram.

Sixty-seven patients were lost to follow-up (dropout rate 23.4%): 31 patients (21.3%) from the IG and 36 (25.5%) from the CG. However, these dropouts did not change the initial characteristics of the study sample. Figure 1 shows the CONSORT flow diagram.

### 3.2 Patients’ baseline characteristics

The 286 study subjects were predominantly male (84.3%), with an average age of 69.8 (95% CI, 69.25–70.43) and a low education level. More than half (58.7%) had been smokers (x = 49.46 packs per year, 95% CI, 28.74–47.36), with 33.2% being active smokers. With respect to COPD, they suffered an average of 1.12 exacerbations in the preceding year (95% CI, 1.02–1.21). The mean pFEV1 was 62.1% (95% CI, 60.85–63.35); the mixed pattern was 56.8%. Furthermore, a large number also had comorbid chronic diseases, at least one, with high blood pressure (HBP) being the most frequent (53.8%). The quality of life was negatively affected.

Table 1 shows the baseline characteristics of the sample per group. The comparison between branches showed significant differences in the use of Turbuhaler^®^ (CG used less; *p* = 0.007), educational level (CG had a higher educational level; *p* = 0.038), the pneumologist’s instructions (CG was better instructed in the IT, *p* = 0.045), and the type of previous instruction for the IT (CG had been previously instructed more through demonstration with the device, *p* = 0.024, and less using explanation with the device, *p* = 0.003).

**TABLE 1:** Baseline sample characteristics and baseline comparison according to the study arm.

Variables	Control group (n = 141)	Intervention group (n = 145)
Sex, n (%), male	123 (87.2)	118 (81.4)
Age (years) [mean, CI 95%]	69.30 (67.70–70.89)	70.36 (68.66–72.06)
Low educational level, n (%)	112 (80)*	128 (88.9)*
Smokers, n (%)	48 (34)	47 (32.4)
Packets/year [mean, CI 95%]	51.03 (45.01–57.04)	47.94 (42.14–53.73)
Comorbidities		
AHT, n (%)	78 (55.3)	76 (52.4)
OP, n (%)	40 (28.4)	53 (36.6)
DM, n (%)	29 (20.6)	35 (24.1)
Diagnostic time (months) [mean, CI 95%]	86.30 (76.45–96.16)	94.46 (83.39–105.52)
COPD pattern, n (%)		
Obstructive	33 (24.6)	27 (19.7)
Restrictive	24 (17.9)	25 (18.2)
Mixed	74 (55.2)	80 (58.4)
COPD severity, n (%)		
Mild	24 (17.8)	26 (19)
Moderate	75 (55.6)	72 (52.6)
Severe	29 (21.5)	37 (27)
FEV1% [mean, CI 95%]	61.30 (57.67–64.93)	62.90 (59.51–66.28)
Inspiratory peak flow [mean, CI 95%]	182.18 (168.42–195.93)	176.42 (165.58–187.26)
Number of exacerbations/year [mean, CI 95%]	1.15 (0.87–1.42)	1.09 (0.82–1.36)
Total visits to HC [mean, CI 95%]	6.22 (5.32–7.12)	6.45 (5.55–7.34)
Visits to HC because of COPD [mean, CI 95%]	2.10 (1.64–2.56)	2.06 (1.68–2.44)
Prescribed treatment, n (%)		
Anticholinergic	92 (67.2)	84 (57.9)
Beta-2 adrenergic	121 (88.3)	131 (90.3)
Inhaled corticosteroids	89 (65)	101 (69.7)
SGRQ [mean, CI 95%]		
Total	33.07 (29.76–36.38)	34.72 (31.76–37.69)
Activities	46.81 (42.72–50.91)	47.16 (43.42–50.90)
Symptoms	41.18 (37.11–45.24)	44.33 (40.68–47.98)
Impact	25.09 (21.88–28.29)	25.50 (22.61–28.38)
EuroQol-5D n (%) with no problems		
Mobility	86 (61)	98 (67.6)
Self-care	119 (84.4)	124 (85.5)
Usual activities	114 (80.9)	118 (81.4)
Anxiety/depression	102 (72.3)	99 (68.3)
Pain/discomfort	78 (55.3)	67 (46.2)
VAS	66.06 (62.07–70.04)	63.57 (59.90–67.25)
BDI, n (%)		
Functional impairment	19 (13.5)	19 (13.1)
Magnitude of task	27 (19.1)	18 (12.4)
Magnitude of effort	28 (19.9)	19 (13.1)
MMRC, n (%)	47 (33.3)	36 (24.8)
MMSE [mean, CI95%]	27.82 (27.32–28.32)*	27.03 (26.5–27.57)*

AHT, arterial hypertension; BDI, baseline dyspnea index; COPD, chronic obstructive pulmonary disease; DM, diabetes mellitus; EuroQol-5D, European Quality of Life-5 Dimensions; VAS, Visual Analog Scale; FEV1, forced expiratory volume in 1 s; HC, health center; MMRC, Modified Medical Research Council Dyspnea Scale; MMST, Mini-Mental Status Examination Test; OP, osteoarticular pathology; RT, randomized trial; SGRQ, St. George Respiratory Questionnaire. *, statistically significant differences (*p* < 0.05).

With regard to the IT, 263 patients (92%) did not perform correctly. The Turbuhaler^®^ was prescribed in 134 (46.9%), the pMDI in 105 (36.7%), the Handihaler^®^ (33.6%) in 96, the Accuhaler^®^ (22.2%) in 64, the Breezhaler (19.6%) in 56, and other inhalers (23.1%) in 66 subjects. Incorrect IT was observed in 120 patients (89.6%) with Turbuhaler^®^, 103 (98.1%) with pMDI, 86 (89.6%) with Handihaler^®^, 50 (78.1%) with Accuhaler^®^, and 50 (78.1%) with Breezhaler^®^. Two hundred and sixty-five patients (93%) had been given some type of IT training, and the average time elapsed from this education to recruitment was 40.09 months (95% CI, 37.41–42.77). GPs carried out the majority of educational training (144 subjects; 50.9%), followed by pulmonologists (99 subjects; 35%). The most common way in which this instruction was performed was through an explanation of the device (124 subjects; 43.7%), followed by demonstration with the device (89 subjects; 31.3%), explanation without the device (31 subjects; 10.9%), and demonstration and explanation without the device (17 subjects; 6%). In four patients (1.4%), the training consisted of handing over a descriptive brochure.

The most common errors, not related to the device, included i) incomplete exhalation before inhaling (84.6%), ii) failure to hold breath or experiencing shortness of breath after inhalation (67.6%), and iii) non-optimal inhaling force (23.7%). These all have moderate clinical significance. The most repeated errors associated with the devices were pressing the button (Turbuhaler^®^ 35.8%, Handihaler^®^ 20.8%, pMDI 23.3%, and Breezhaler^®^ 7.3%) and shaking the pMDI device (52.4%). Table 2 sets out the baseline characteristics of the IT per device and CSF.

**TABLE 2:** Inhalation technique by type of device.

Incorrect IT n (%)										
Handihaler®		Accuhaler®		Turbuhaler®		Breezhaler®		pMDI		
BV	FV	BV	FV	BV	FV	BV	FV	BV	FV
86 (89.6)	31 (64.6)	50 (78.1)	21 (48.8)	120 (89.6)	61 (58.7)	50 (89.3)	32 (62.7)	103 (98.1)	50 (60.2)	
Most frequent mistakes										
No full exhale before inhalation n (%)										
CSF n (%)										
78 (81.3)	30 (62.5)	51 (78.5)	20 (46.5)	114 (85.1)	59 (56.7)	47 (83.9)	28 (56)	86 (82.7)	43 (51.8)	
CSF2: 78 (100)	CSF2: 30 (100)	CSF2: 51 (100)	CSF2: 20 (100)	CSF2: 114 (100)	CSF2: 59 (100)	CSF2: 47 (100)	CSF2: 28 (100)	CSF1: 86 (100)	CSF1: 43 (100)	
Not pushing the button correctly n (%)										
CSF n (%)										
20 (20.8)	8 (16.7)	7 (10.9)	2 (4.7)	48 (35.8)	8 (7.6)	4 (7.3)	9 (18)	24 (23.3)	10 (12)	
CSF2: 15 (75)	CSF2: 8 (100)	CSF3: 7 (100)	CSF3: 2 (100)	CSF3: 48 (100)	CSF3: 8 (100)	CSF1: 1 (25)	CSF2: 9 (100)	CSF2: 6 (25)	CSF2: 3 (30)	
CSF3: 5 (25)						CSF2: 2 (50)		CSF3: 18 (75)	CSF3: 7 (70)	
						CSF3: 1 (25)				
Not placing lips correctly on the mouthpiece n (%)										
CSF n (%)										
2 (2.1)	0	2 (3.1)	0	2 (1.5)	0	0	0	13 (12.6)	6 (7.2)	
CSF1: 1 (50)		CSF2: 2 (100)		CSF2: 1 (50)				CSF1: 3 (23.1)	CSF3: 6 (100)	
CSF3: 1 (50)				CSF3: 1 (50)				CSF2: 6 (46.2)		
								CSF3: 4 (30.8)		
Non-optimal strength of inhalation n (%)										
CSF n (%)										
8 (8.3)	1 (2.1)	7 (10.8)	0	11 (8.2)	2 (1.9)	3 (5.5)	0	72 (69.9)	24 (28.9)	
CSF1: 5 (62.5)	CSF1: 1 (100)	CSF1: 3 (42.9)		CSF1: 2 (18.2)	CSF2: 2 (100)	CSF1: 1 (33.3)		CSF2: 72 (100)	CSF2: 24 (100)	
CSF2: 3 (37.5)		CSF2: 4 (57.1)		CSF2: 7 (63.6)		CSF2: 2 (66.7)				
				CSF3: 2 (18.2)						
No or short breath hold after inhalation n (%)										
CSF n (%)										
65 (67.7)	18 (37.5)	40 (61.5)	14 (32.6)	81 (60)	33 (31.7)	40 (72.7)	22 (44)	77 (74.8)	33 (39.8)	
CSF2: 65 (100)	CSF2: 18 (100)	CSF2: 40 (100)	CSF2: 14 (100)	CSF2: 81 (100)	CSF2: 33 (100)	CSF2: 40 (100)	CSF2: 22 (100)	CSF2: 77 (100)	CSF2: 33 (100)	

BV: baseline visit; CSF: clinical significance of the failure: CSF1: mild, CSF2: moderate, CSF3: critical error; FV: final visit; IT: inhalation technique.

### 3.3 GPs’ baseline characteristics

The 27 GPs had an average age of 55.64 years (CI 95%, 56.62–54.66), and 59.3% were women. The majority (91.7%) were family and community medicine specialists and 9.3% had completed their doctoral studies. About the last update on COPD, 41.7% had attended clinical sessions and reviewed national recommendations; 33.3% had taken courses; 20.8% had received other types of updates; 12.5% had reviewed international recommendations; and 4.2% had attended congresses.

Regarding the COPD guidelines, 62.5% knew the national guidelines (the Spanish COPD guide, GesEPOC, Integrated Care Process of COPD) and 37.5% knew the international guidelines (the Global Initiative for COPD, GOLD). As a result of the test of knowledge about COPD diagnosis and management, no professional answered the full questionnaire correctly. If we split the questionnaire into questions related to the diagnosis, classification, and management of COPD, we found the correct answers in 3 (11%), 7 (26%), and 2 (7%) GPs, respectively.

Concerning IT, 91.7% (25) of the professionals performed it incorrectly, 92.3% in the IG and 92.9% in the CG. Incorrect IT was detected in 19 subjects (79.28%) with Turbuhaler^®^, 19 (86.4%) with pMDI, 22 (91.7%) with Handihaler^®^, 17 (70.8%) with Accuhaler^®^, and 20 (90.9%) with Breezhaler^®^.

Twenty GPs (74.4%) had been given some type of IT training, and the average time elapsed from this education to recruitment was 30.2 months (95% CI, 6.96–58.84). The pulmonologist carried out the majority of educational training (seven GPs; 25.9%), followed by Big Pharma courses (three GPs; 11.1%) and healthcare service courses (three GPs; 11.1%). The most frequent way in which this instruction was performed was through the demonstration with the inhaler (15 subjects; 55.6%), followed by instruction with device explanation (5 subjects; 18.5%), and in one GP (3.7%), the instruction consisted of a descriptive brochure.

The most recurrent errors, observed and not related with the device, included failure to hold breath or experiencing shortness of breath after inhalation (60.9%) and incomplete exhalation before inhaling (36.2%), with moderate clinical significance. The most frequent errors related to the inhalers were correct position for Turbuhaler^®^ (16.4%), emptying the content for Handihaler^®^ (75%) and Breezhaler^®^ (77.3%), and coordination for pMDI (60%).

Regarding the review of the IT with their patients, 100% agree that it should be reviewed periodically: when the device is changed (41.7%), at the beginning of treatment (37.5%), at each consultation or each year (25%), when the patient requests it (12.5%), or every 6 months (4.2%).

GPs reported that they tended to review the IT in COPD patients when introducing a new device (79.2%), only once (16.7%), never (12.5%), or every 3 months (4.2%).

### 3.4 Intervention effectiveness

**FIGURE 2:**
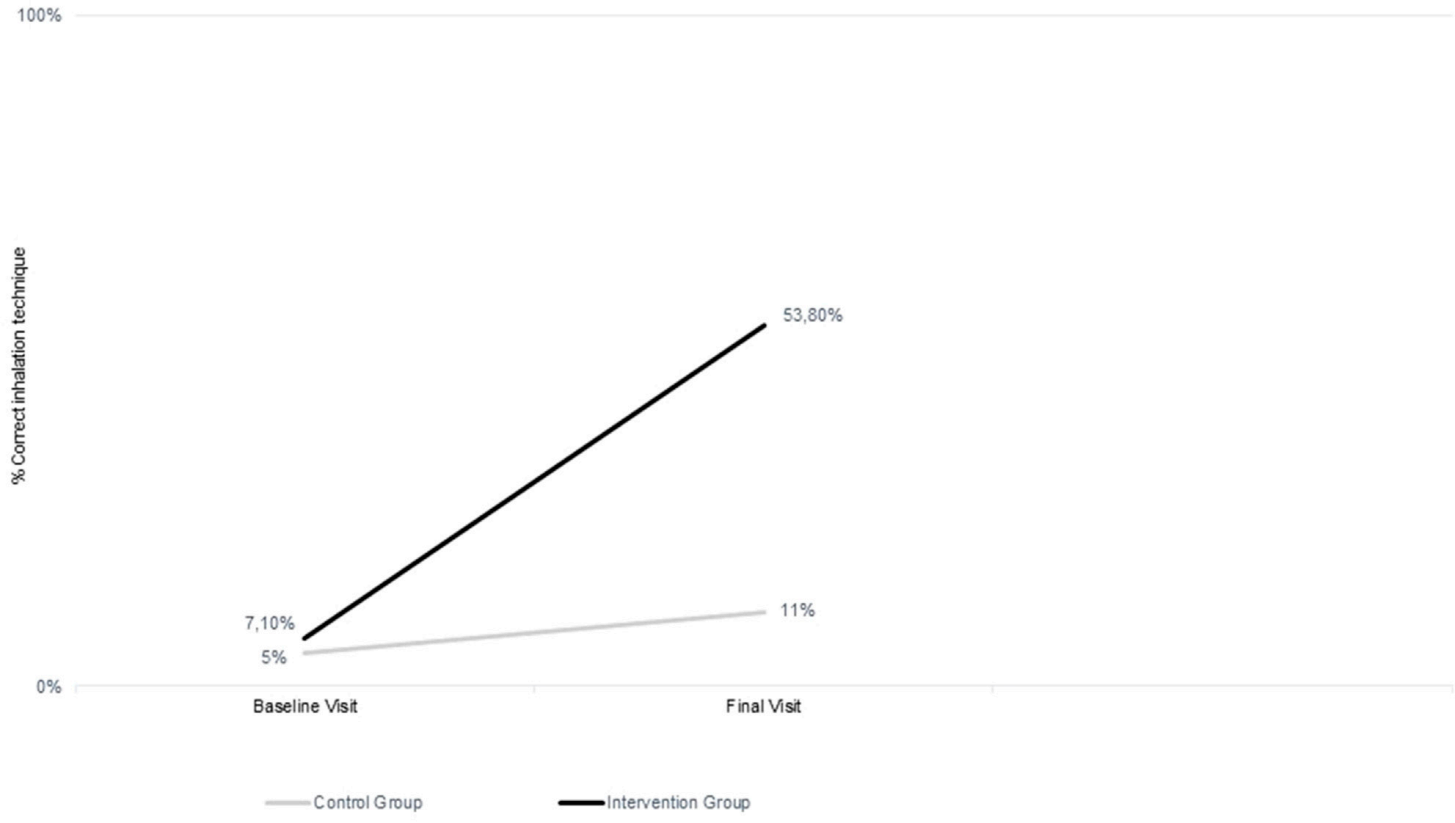
Evolution of correct inhalation technique.

On finishing the study, the success IT rates were found to be 78 subjects (53.8%) for the IG and 10 subjects (7.1%) for the CG (*p* < 0.0001). Figure 2 shows the progression of the IT in both groups.

The effectiveness parameters calculated for this study were RR = 7.58 (CI 95%, 4.09–14.04), ARB = 6.57, AAB = 0.46 (95% CI, 0.37–0.46), and NNT = 2.14 (95% CI, 1.79–2.66), which means that, for every 2–3 patients who are trained by their GP, an additional clinical benefit is achieved (correct IT).

The mean time for device educational training was 5.21 min (CI 95%, 4.98–5.44) for the CG and 5.98 min (CI 95%, 5.49–6.47) for the IG at baseline. On finishing the study, it was 5.21 min (CI 95%, 4.76–5.66) for the CG and 5.98 min (CI 95%, 5.01–6.95) for the IG.

For the step-by-step performance of the IT and also by devices, the comparison between baseline and the end of the study is summarized in Table 2.

For the GPs, the comparison between baseline and the final visit showed statistically significant differences for the correct performance of the IT in general and by devices for the IG. For the general IT, there was an improvement of 61.5% (*p* < 0.0001). No statistically significant differences were found for any other variable in either the IG or CG.

The comparison of secondary outcomes (patients) at the end of the trial is set out in Table 3. Statistically significant differences are shown for the VAS scale of EuroQoL-5D-5L, with better perceived health status in both groups (CG *p* = 0.028; IG *p* < 0.0001). Moreover, statistically significant differences were found for the SGRQ total scale (*p* = 0.041) and SGQR symptom scale (*p* < 0.0001) scales in the IG.

**TABLE 3:** Comparison between secondary outcome variables BV and FV.

Variables	Control group			Intervention group		
	BV (n = 141)	FV (n = 145)	*p*	BV (n = 141)	FV (n = 145)	*p*
EuroQol, n (%)						
Self-care problems	22 (15.6)	27 (25.8)	*0.113*	21 (14.5)	17 (14.9)	*0.940*
Usual activities problems	27 (19.1)	*32 (30.5)*	*0.117*	27 (18.7)	21 (19.3)	*0.554*
Pain/discomfort	63 (44.6)	56 (53.4)	*0.395*	78 (53.8)	50 (43.8)	*0.244*
Anxiety/depression	39 (27.6)	40 (38.1)	*0.092*	46 (31.8)	*30 (26.3)*	*0.244*
EuroQol VAS [mean, CI 95%]	66.06 (62.07–70.04)	72.29 (68.69–75.88)	0.028*	63.57 (59.90–67.25)	72.37 (69.12–75.61)	0.001*
SGRQ [mean, CI 95%]						
Total	34.32 (31.08–37.56)	34.17 (30.01–38.33)	*0.955*	35.48 (32.58–38.38)	31.07 (27.92–34.22)	0.044*
Symptoms	42.73 (38.75–46.71)	38.73 (34.03–43.43)	*0.199*	45.29 (41.73–48.85)	34.76 (30.96–38.55)	0.000*
Activities	46.81 (42.72–50.91)	46.86 (41.42–52.30)	*0.989*	47.16 (43.42–50.90)	43.22 (39.44–47.01)	*0.150*
Impact	25.09 (21.88–28.79)	25.44 (21.50–29.39)	*0.899*	25.50 (22.61–28.38)	22.35 (18.96–25.74)	*0.161*
Dyspnea, n (%)						
BDI functional impairment	122 (86.5)	122 (86.5)	*0.569*	126 (86.9)	126 (86.9)	*0.569*
BDI magnitude of task	114 (80.9)	114 (80.9)	*0.560*	127 (87.6)	127 (87.6)	*0.571*
BDI magnitude of effort	113 (80.1)	113 (80.1)	*0.559*	126 (86.9)	126 (86.9)	*0.569*
MMRc scale	47 (33.3)	36 (24.8)	*0.550*	47 (33.3)	36 (24.8)	*0.554*
FEV1% [mean, CI 95%]	61.3 (57.71–64.89)	62.9 (59.64–66.16)	*0.091*	56.73 (53.61–59.85)	64.38 (60.82–67.94)	*0.552*
Inspiratory peak flow [mean, CI95%]	182.18 (168.75–197.59)	176.42 (165.67–187.17)	*0.484*	175.19 (163.60–186.78)	185 (172.51–197.49)	*0.371*
COPD severity, n (%)			*0.393*			*0.821*
Mild	24 (17)	26 (17.9)		17 (12.1)	24 (16.6)	
Moderate	75 (53.2)	72 (49.7)		45 (31.9)	57 (39.3)	
Severe	36 (25.6)	39 (26.9)		36 (25.6)	26 (18)	

BDI, basal dyspnea index; BV, baseline visit; VAS, Visual Analogic Scale; FV, final visit; MMRc, Modified Medical Research Council Dyspnea Scale; SGRQ, Saint George Respiratory Questionnaire. The *p*-values are the marked in italic.

### 3.5 Intervention-related factors

Table 4 summarizes the multivariate analyses. A logistic regression analysis and a classification tree analysis were performed as multivariate analyses for this study. Both analyses showed the same results; so finally, classification tree analysis was chosen to describe the results for this paper. Several analyses were performed with and without missing data, but the results were the same. In addition, the losses were less than those calculated for sample size, so it was decided to exclude dropouts from the analysis in order to avoid statistical artifacts from this cause.

**TABLE 4:** Factors associated with the correct inhalation technique at the final visit.

Variables	% correct IT	*p*	Significance
Intervention group vs. control group	53.8 vs. 7.1	0.0001*	Intervention group shows a better IT
Explanation with a device vs. others	72.1 vs. 48.5	0.002*	IT improves using explanation with a device
pFEV	77.3 vs. 56.5	0.0001*	Better pulmonary function improves the IT
SGRQ total scale	0 vs. 14.7	0.013*	Having a better quality of life improves the IT
SGRQ activity scale	65.9 vs. 75	0.0001*	
Intervention time ≥25 min	77.4 vs. 55.5	0.0001*	IT improves if the interview is ≥25 min

IT: inhalation technique; SGRQ, Saint George Respiratory Questionnaire. The *p*-values are the marked in italic.

With the classification tree analysis, it was observed that membership in the intervention group discriminated in favor of a better IT (53.8% IG vs 7.1% CG, *p* < 0.0001). Furthermore, the explanation with the device improved the performance of the IT (0% IG vs. 72.1% CG, *p* = 0.002). In the patients of the IG, good pulmonary function favored the good performance of the technique (56.5% vs. 77.3%, *p* = 0.0001). Likewise, among those with poorer pulmonary function, age influenced good performance (*p* = 0.014), with the youngest patients (≤65) and the oldest (>83) returning the best results, while the intermediate age group had the lowest percentage of good performance (34.5% vs. 90% and 100%).

A good IT is inversely associated with the quality of life in the CG regarding the total SGRQ scale (*p* = 0.013). For the IG, a relationship was found between a lower limitation of activity due to dyspnea (activity scale of SGRQ, *p* < 0.0001) and a good performance of the IT. Finally, an intervention lasting more than 25 min significantly improves the performance of a good IT (55.1 vs. 77.4, *p* < 0.0001). No relation was found between the prior IT trainer and dyspnea (BDI and mMRC).

With regard to the GPs, there was no influence of age, sex, previous IT performance, or knowledge about COPD and its treatment. Only membership in the intervention group is related to a better IT performance.

## 4 Discussion

The PROF-EPOC study shows that an educational intervention using the teach-back method with GPs has a significant and positive effect on GPs’ performance of the IT and that of their patients after 1 year of follow-up. This improvement was associated not with the characteristics of the GP but with the intervention. This study found that good performance of the IT was related to demonstrating the proper technique with the device, having a good pulmonary function, being among the youngest (≤65) and the oldest (>83) patients, experiencing lower limitation of activity due to dyspnea, and undergoing an intervention lasting more than 25 min. This educational intervention involving GPs could be a promising approach to improving medication adherence and health outcomes in COPD patients.

There are few studies addressing interventions focused on the training of healthcare professionals on the IT, and fewer focused on GPs or COPD patients. Toumas et al. (2009) conducted a study with second-year pharmacy students who were given a brochure and found that 10% of the participants performed the technique properly as a result. However, when they were given a demonstration with the device, this percentage increased to 62%. Al-Otaibi (2020) performed the intervention on physicians, pharmacists, respiratory therapists, and health educators who attended a training course where an improvement in the inhaler handling questionnaire score of almost 40% was observed. Cvetkovski et al. (2020) carried out their study on primary care physicians, showing that only 0.88% of the professionals performed the IT incorrectly after reading a leaflet and witnessing an explanation with a device. The present study showed a similar increase for the proper IT for GPs.

Regarding the improvement of the IT in patients after the training of their GPs, there are few studies in the literature carried out on COPD patients, and most are conducted jointly with asthma patients. Aksu et al. (2016) conducted a prospective study of 108 patients with asthma and COPD. The physician made an initial visit where the patient’s IT was corrected, and after 3 months, the IT was checked again. The percentage of patients with good IT improved (28%), showing how the practical training provided by physicians on the management of inhalers was an effective tool in the improvement of ITs. Takaku et al. (2017) carried out a prospective observational study with 216 subjects with asthma (the majority) and COPD. The counseling was performed by a pharmacist who had received prior training. They reported an improvement of 53% in the IT after the intervention. Takemura et al. (2013) performed a study training 81 community pharmacists to educate patients through a brochure. A review was conducted 4 years later, and it was observed that 39 patients had improved adherence and quality of life, including the IT. These studies show that training HCP improves the IT in their patients, in line with the results of our own study. However, these studies did not include effectiveness parameters. Only the study of Kim et al. (2021) reported an NNT of 3.3, which is similar to our result. Further studies are, thus, necessary to address this topic.

There is apparent agreement on the need to demonstrate the practical management of inhalers by professionals before prescribing them. The IT should be performed appropriately during every appointment at the healthcare center and should be controlled by healthcare professionals (Aksu et al., 2016; Lavorini et al., 2019; Melani, 2021; Global Iniciative for COPD, 2022). When a change in treatment takes place, a new demonstration must be repeated by both the healthcare professional and the patient (Dekhuijzen et al., 2022). Similar results were obtained in the present study by questioning GPs. The IT must also be reviewed periodically in order to be effective (Kim et al., 2021; Dekhuijzen et al., 2022; Global Iniciative for COPD, 2022), as a relationship between regular instruction and adherence has been observed (Martínez Ibán et al., 2019; Ahn et al., 2020; Efil et al., 2020), with an improvement in the quality of life (Martínez Ibán et al., 2019; Efil et al., 2020; Luley et al., 2020; Lindh et al., 2022) and a reduction in the need for hospital admission of up to 80% (Martínez Ibán et al., 2019; Efil et al., 2020). Thus, the professionals considered that the IT should be checked periodically, although they did not agree on how often it should be checked. However, previous studies carried out in the same environment showed that reminders about the IT for COPD patients should take place every 3 months (Barnestein-Fonseca et al., 2023). Other studies suggest the same frequency of 3 months improves IT and adherence (Takaku et al., 2017; Ahn et al., 2020), while another recommends a lesser frequency (Lee et al., 2016; Yoo et al., 2017).

It is clear that suitable instruction can improve IT (Klijn et al., 2017; Barnestein-Fonseca et al., 2023). However, due to varying educational levels, different teaching techniques are used. Essentially, these teaching techniques can be classified into two types: brochures and practical demonstrations. The studies of educational IT interventions (Toumas et al., 2009; Bosnic-Anticevich et al., 2010; Klijn et al., 2017; Jia et al., 2020; Melani, 2021; Barnestein-Fonseca et al., 2023) showed that interventions including a face-to-face or video demonstration showing how to use inhalers are effective. In the same way, after testing which educational interventions were the most appropriate for COPD patients in our environment, it was decided to include an educational intervention based on a practical demonstration of the IT (Barnestein-Fonseca et al., 2011; Barnestein-Fonseca et al., 2023).

Our work shows fewer IT improvements than most studies carried out, although there are some exceptions (Giner et al., 2002; Cabedo García et al., 2010; O’Dwyer et al., 2020). This may be because the analysis was performed considering the intention-to-treat principle, while the other authors did not take into account the dropouts.

Although many patients indicated that they had received IT instruction, the rate of incorrect techniques was high. This fact could be due, in part, to the limited knowledge of the professionals who prescribe these medicines on how to manage inhalers and the teaching techniques (Aksu et al., 2016; Plaza et al., 2018; Al-Otaibi, 2020; Cvetkovski et al., 2020), as well as to a lack of regular IT verification with reminders and to the kind of training chosen (Klijn et al., 2017; Takaku et al., 2017; Kaplan and Price, 2018; Lavorini et al., 2019; Melani, 2021; Lindh et al., 2022). These results agree with the findings of this work, where the GPs manifested a high level of incorrect IT in their patients and where the reminders of the IT were performed with inappropriate periodicity.

In addition, it is essential to consider that reminders are important not only for patients but also for professionals so that the training they provide to their patients will also be correct. Some authors claim that GPs may be served by less frequent updating of skills in the management of particular inhalers compared to others (Takemura et al., 2011; Bosnic-Anticevich et al., 2018; Cvetkovski et al., 2020). Although regular educational interventions do indeed improve the long-lasting consistency of the IT (Takemura et al., 2011), from the perspective of GPs, illness management should be prioritized (Cvetkovski et al., 2020). It is clearly demonstrated that motivation plays an essential role in the IT, as it is not just a physical skill (Bosnic-Anticevich et al., 2018). Whether GPs have the time and disposition to educate COPD patients about the IT and implement strategies to improve adherence and proper use of medicines remains unanswered (Cvetkovski et al., 2020).

Several works indicated that from 50% to 94% of patients are not able to carry out the inhalation technique correctly (Chrystyn et al., 2017; Adib-Hajbaghery and Karimi, 2018; Duarte-de-Araújo et al., 2019; Rincon-Montaña and Rosselli, 2019; Dhadge et al., 2020; Vila Jato, 2020; Dekhuijzen et al., 2022; Sulku et al., 2022), even though the success of the treatment depends on it (Chrystyn et al., 2017; Adib-Hajbaghery and Karimi, 2018; Duarte-de-Araújo et al., 2019; Lindh et al., 2019; Padmanabhan et al., 2019; Rincon-Montaña and Rosselli, 2019; Rodriguez-Garcia et al., 2020; Schreiber et al., 2020; Dekhuijzen et al., 2022). The errors found in all the devices are similar to those reported by Melani (2021) in a review. Previous works revealed that the errors depended on the subject’s preparedness and physical ability to execute the technique. The most common errors found were to achieve lower peak inhalation flow, lower MMSE scores, fewer appointments with the pulmonologist, and not receiving previous educational management of inhalers (Leiva-Fernandez et al., 2013; Barnestein-Fonseca et al., 2022). Errors associated with inhalers are less frequent and are linked to different positions (in the case of Turbuhaler^®^) and flows (coordination in the case of pMDI) (Chrystyn et al., 2017; Duarte-de-Araújo et al., 2019; Lindh et al., 2019). Despite improvements and breakthroughs in technology, most subjects do not intuitively reach full competence with the inhaler by themselves (Harb et al., 2020; Melani, 2021). Studies using real-world data inform us that, as of yet, there is no easy-to-use inhaler available.

A poor IT leads to a reduction of its beneficial effect, lower symptom control, and therefore, poor COPD control (Padmanabhan et al., 2019; Vila Jato, 2020; Dekhuijzen et al., 2022). It may also be associated with an increase in the number of emergency room visits, hospitalizations, or the use of antibiotic and corticosteroid treatments, ultimately increasing the cost of COPD management, increasing adverse reactions, and limiting therapeutic alternatives (Martínez Ibán et al., 2019; Efil et al., 2020; Vila Jato, 2020; Dekhuijzen et al., 2022). The performance of the correct IT has been related, in this study as well, to a better FEV1, perhaps because correct performance allows for better FEV1, potentially slowing down functional deterioration, and it then improves the technique because there are steps that are related to good pulmonary function, such as deep inhalation of the aerosol (Melani et al., 2011; Padmanabhan et al., 2019; Efil et al., 2020; Vila Jato, 2020). Among those with poorer lung function, the percentage of those performing the correct IT was influenced by age; specifically, those under 65 and over 83 years of age demonstrated a higher percentage of correct technique. These findings could be associated with a greater concern for the progression of COPD in younger and older people with shorter and longer diagnosis times, respectively. It has been found that the performance of the correct IT is related to QoL measured by SGRQ in both groups. In the CG, the modification is observed at the global scale, and there is a higher percentage of subjects with the correct technique among those who have a poorer quality of life. This could be explained by the fact that, feeling worse, they make a greater effort to take advantage of the benefits that the treatments can provide for them. In the IG, the differences are observed on the activity scale. The subjects with higher scores (bad quality of life) perform the technique less well than those with lower scores. Poor performance of the technique makes it difficult to perform daily activities (Melani et al., 2011; Padmanabhan et al., 2019).

Finally, when the visit lasts longer than 25 min, there is a higher percentage of patients who perform well on the IT (Weheida et al., 2017; Efil et al., 2020; Lindh et al., 2022). This finding correlates with the methods used for training the patients. The intervention includes the feedback of the patients until they develop a correct IT, so to obtain a higher percentage of good results, it is necessary to spend time on correct training. However, when the training is fixed, the time of intervention decreases during the follow-up visits (Kim et al., 2021; Barnestein-Fonseca et al., 2023).

Overall IT performance for GPs showed the same errors as the patients, those related to their preparation before performing the technique. However, when analyzed by devices, the most frequent errors among GPs are related to the device itself, even if the type of error is the same. This is perhaps because they do not have lung capacity problems, as their patients do. The consistency in the type of error over different devices is understandable, given that they have the same characteristics (Dekhuijzen et al., 2022). However, are the errors subject related rather than device related? Could there be a transfer of knowledge between inhalers (Dekhuijzen et al., 2022)? This was partially noted in this study, with GP errors related to their preparation for the technique. Thus, focusing education on the most common known errors could help improve full IT competence, regardless of the inhaler used.

The main strength of this study is the use of an intervention that is quick, easy, and reproducible to improve the IT, based on the practical demonstration of the IT using the teach-back method, previously tested in clinical trials (Barnestein-Fonseca et al., 2011; Barnestein-Fonseca et al., 2023). With these methods, patients can demonstrate their inhaler handling and subsequently receive specific feedback from the instructor. In addition, the dropout percentage was found to be lower than expected, and there were no differences between the initial and final samples. Therefore, the internal validity of the study is guaranteed.

This work also has limitations. First, the missing data lead to a loss of estimation accuracy. To address this, the sample size was increased by 40% (i.e., the expected losses), telephone calls were made to unreachable patients at different times, and extra appointments for clinic visits were scheduled; these resulted in lower losses than expected. Moreover, we applied data imputation to complete the lost data. Second, randomization was applied at the second level before the recruitment of participants from the first level, where the impact of the intervention was measured, which could lead to selection bias at this level. To minimize this bias, external research assumed the patients’ selection and their follow-up; this person did not know the GP randomization. In addition, different motivations among randomized professionals could lead to different recruitment rates, as control GPs may be less willing to cooperate. To counteract this lack of motivation, control professionals received the same training as intervention professionals at the end of the trial (Basheti et al., 2008; Ahn et al., 2020). Another potential bias could have come from the selective correction of only critical errors in the control group patients, which could have worked in favor of the study hypothesis by increasing the rate of the incorrect technique in this group, considering that all the steps indicated in the template had to be fulfilled in the assessment of the technique. Another limitation could be interviewer bias in the measurement of variables based on the application of questionnaires due to the involvement of different interviewers in administering them. To minimize these biases, the interviewer monitors were previously trained to ensure that the visits were as homogeneous as possible. To avoid this bias among the health professionals in the intervention group, who were, therefore, in charge of training their patients in the correct inhalation technique, they were thoroughly trained and provided with a common data collection booklet and an explanatory manual on how to collect each of the variables that were measured in the follow-up visits of their patients.

The results of this research could have a major impact on the prognosis of the disease, making it a promising approach. This is an easy-to-implement intervention with high potential for real-life efficacy in improving medication adherence and health outcomes in COPD patients. As a recommendation for implementation in the clinical setting, this training could be extended to all professionals involved in the care of COPD patients; it would represent a more effective strategy that could benefit a larger number of individuals. It is also important to consider changing the inhaler used or applying a spacer, especially for those who have greater difficulties handling the different devices due to their age and physical and/or mental disability.

As a group of patients still struggled to manage their inhalers, a more detailed analysis of patient characteristics would be necessary to modify certain phases of the training (e.g., frequency of reminders for both professionals and patients). In conclusion, this study shows the efficacy of direct training using the teach-back method in the inhaler technique in patients by a trained professional (general practitioner) with sufficient time (e.g., specific consultation for medication review).

## Data Availability

The raw data supporting the conclusion of this article will be made available by the authors, without undue reservation.
