# Supplementary material for: Effectiveness of an educational intervention about inhalation technique in healthcare professionals in primary care: a cluster randomized trial
Source: Front Pharmacol. 2023 Oct 17;14:1266095. doi: 10.3389/fphar.2023.1266095 (PMC10617029; doi:10.3389/fphar.2023.1266095)
Supplement: Supplementary file 1 [file Table1.DOCX]

**Supplementary material 1. Ad hoc step-by-step template for Inhalation Devices**

Each of the steps of the 5 devices included in the study are recorded: Handihaler®, Turbuhaler®, Accuhaler®, Metered Dose Inhalers and Breezhaler®.

| **Handihaler® y Breezhaler®** | **Error** | **CSF** | **Comments** |
| --- | --- | --- | --- |
| 1.- Open device |  |  |  |
| 2.- Place the capsule vertically |  |  |  |
| 3.- Mouthpiece upwards and press the button |  |  |  |
| 4.- Breath out slowly and deeply, avoiding breathing out over the device. |  |  |  |
| 5.- Mouthpiece between teeth and closed lips |  |  |  |
| 6.- Inhale vigorously and deeply |  |  |  |
| 7.- Hold your breath for about 10 seconds |  |  |  |
| 8.- Emptying the content of the device |  |  |  |
| 9.- Close the inhaler |  |  |  |

| **Accuhaler® y Turbuhaler®** | **Error** | **CSF** | **Comments** |
| --- | --- | --- | --- |
| 1.- Open device and press trigger |  |  |  |
| 2.- Breathe out slowly and deeply, avoiding breathing out over the device. |  |  |  |
| 3.- Put lips on the mouthpiece |  |  |  |
| 4.- Inhale vigorously and deeply |  |  |  |
| 5.- Hold your breath for about 10 seconds |  |  |  |

| **Meterd Dose Inhalers** | **Error** | **CSF** | **Comments** |
| --- | --- | --- | --- |
| 1.- Shake the device |  |  |  |
| 2.- Breathe out slowly and deeply |  |  |  |
| 3.- Place the cartridge mouthpiece between the lips |  |  |  |
| 4.- Keep the device in the vertical position |  |  |  |
| 5.- Inhale slowly and deeply |  |  |  |
| 6.- Press cartridge |  |  |  |
| 7.- Keep inspiring |  |  |  |
| 8.- Hold your breath for about 10 seconds |  |  |  |

(SEPAR, 2013); CSF: Clinical Significance of Failure (Melani, 2021).
