# Supplementary material for: Effectiveness of an educational intervention about inhalation technique in healthcare professionals in primary care: a cluster randomized trial
Source: Front Pharmacol. 2023 Oct 17;14:1266095. doi: 10.3389/fphar.2023.1266095 (PMC10617029; doi:10.3389/fphar.2023.1266095)
Supplement: Supplementary file 2 [file Table2.DOCX]

**Supplementary material 2: ST. GEORGE’S RESPIRATORY QUESTIONNAIRE** (Ferrer et al., 2002)

To complete this test, each item is read to the patient and the option chosen by the patient is marked with a cross.

The questionnaire consists of a total of 50 items (76 levels) divided into three dimensions: Symptoms, Activity and Impact. The "Symptoms" items refer to the frequency and severity of respiratory symptoms. The "Activity" dimension refers to activity limitation due to dyspnoea. The "Impact" dimension contains items referring to the psychological and social functioning impairment caused by the respiratory disease. The items are presented in 2 different forms: a question with a maximum of 5 response options, of which only one must be chosen; or in the form of two-choice sentences, "yes/no"(Ferrer et al., 1997).

The questionnaire items are assigned weights that were obtained from 6 countries: England, Finland, the Netherlands, Italy, Thailand and the USA. Age, gender, demographic or disease-related factors had minimal influence on the weights and thus proved to be appropriate for a wide range of patients with asthma or COPD. EPOC.

A score can be calculated for each of the scales of the questionnaire and also an overall score. For this purpose, the weights corresponding to the positively answered items of each scale are added up, multiplied by 100 and divided by the sum of the weights of all the items of the corresponding scale. The range of possible scores goes from 0 (no impairment of quality of life) to 100 (maximum impairment of quality of life).) (Ferrer et al., 2002).

**ST. GEORGE’S RESPIRATORY QUESTIONNAIRE**

*This questionnaire is designed to help us learn much more about how your breathing is troubling you and how it affects your life.*

*We are using it to find out which aspects of your illness cause you most problems, rather than what the doctors and nurses think your problems are.*

*Please read the instructions carefully and ask if you do not understand anything. Do not spend too long deciding about your answers. Please select ONE box for each question:*

**Questions about how much chest trouble you have.**

**Question 1.** I cough:

- most days a week
- several days a week
- only with chest infections
- not at all

**Question 2.** I bring up phlegm (sputum):

- most days a week
- several days a week
- only with chest infections
- not at all

**Question 3.** I have shortness of breath:

- most days a week
- several days a week
- not at all

**Question 4.** I have attacks of wheezing:

- most days a week
- several days a week
- a few days a month
- only with chest infections
- not at all

**Question 5.** How many attacks of chest trouble did you have during the last year?

- 3 or more attacks
- 1 or 2 attacks
- none

**Question 6.** How often do you have good days (with little chest trouble)?

- no good days
- a few good days
- most days are good
- every day is good

**Question 7.** If you have a wheeze, is it worse in the morning?

- no
- yes

**Question 8.** How would you describe your chest condition?

- Causes me a lot of problems or is the most important problem I have
- Causes me a few problems
- Causes no problem

**Question 9.** Questions about what activities usually make you feel breathless. For each statement please select the box that applies to you these days:

|  | True | False |
| --- | --- | --- |
| Getting washed or dressed |  |  |
| Walking around the home |  |  |
| Walking outside on the level |  |  |
| Walking up a flight of stairs |  |  |
| Walking up hills |  |  |

**Question 10.** Some more questions about your cough and breathlessness. For each statement please select the box that applies to you these days:

|  | True | False |
| --- | --- | --- |
| My cough hurts |  |  |
| My cough makes me tired |  |  |
| I am breathless when I talk |  |  |
| I am breathless when I bend over |  |  |
| My cough or breathing disturbs my sleep |  |  |
| I get exhausted easily . |  |  |

**Question 11.** Questions about other effects that your chest trouble may have on you. For each statement please select the box that applies to you these days:

|  | True | False |
| --- | --- | --- |
| My cough or breathing is embarrassing in public |  |  |
| My chest trouble is a nuisance to my family, friends or neighbours |  |  |
| I get afraid or panic when I cannot get my breath |  |  |
| I feel that I am not in control of my chest problem |  |  |
| I have become frail or an invalid because of my chest |  |  |
| Exercise is not safe for me |  |  |
| Everything seems too much of an effort |  |  |

**Question 12.** These are questions about how your activities might be affected by your breathing. For each statement please select the box that applies to you because of your breathing:

|  | True | False |
| --- | --- | --- |
| I take a long time to get washed or dressed |  |  |
| I cannot take a bath or shower, or I take a long time |  |  |
| I walk slower than other people, or I stop for rests |  |  |
| Jobs such as housework take a long time, or I have to stop for rests |  |  |
| If I walk up one flight of stairs, I have to go slowly or stop |  |  |
| If I hurry or walk fast, I have to stop or slow down |  |  |
| My breathing makes it difficult to do things such as walk up hills, carrying things up stairs, light gardening such as weeding, dance, play bowls or play golf |  |  |
| My breathing makes it difficult to do things such as carry heavy loads, dig the garden or shovel snow, jog or walk at 5 miles per hour, play tennis or swim |  |  |

**Question 13.** We would like to know how your chest trouble usually affects your daily life. For each statement please select the box that applies to you because of your breathing:

|  | True | False |
| --- | --- | --- |
| I cannot play sports or games |  |  |
| I cannot go out for entertainment or recreation |  |  |
| I cannot go out of the house to do the shopping |  |  |
| I cannot do housework |  |  |
| I cannot move far from my bed or chair |  |  |

**Question 14.** How does your chest trouble affect you? Please select ONE:

- It does not stop me doing anything I would like to do
- It stops me doing one or two things I would like to do
- It stops me doing most of the things I would like to do
- It stops me doing everything I would like to do

*Thank you for filling in this questionnaire.*
